# Stability of Monoclonal Antibodies as Solid Formulation for Auto-Injectors: A Pilot Study

**DOI:** 10.3390/pharmaceutics15082049

**Published:** 2023-07-30

**Authors:** Fatima Garcia-Villen, Idoia Gallego, Myriam Sainz-Ramos, Jorge Ordoyo-Pascual, Sandra Ruiz-Alonso, Laura Saenz-del-Burgo, Conor O’Mahony, Jose Luis Pedraz

**Affiliations:** 1 NanoBioCel Group, Laboratory of Pharmaceutics, Faculty of Pharmacy, University of the Basque Country UPV/EHU, 01006 Vitoria-Gasteiz, Spainlaura.saenzdelburgo@ehu.eus (L.S.-d.-B.); 2 Biomedical Research Networking Centre in Bioengineering, Biomaterials and Nanomedicine (CIBER-BBN), Institute of Health Carlos III, 01006 Vitoria-Gasteiz, Spain; 3 Bioaraba, NanoBioCel Resarch Group, 01009 Vitoria-Gasteiz, Spain; 4 Tyndall National Institute, University College Cork, T12 R5CP Cork, Ireland; conor.omahony@tyndall.ie

**Keywords:** monoclonal antibody, stability, nano-DSC, solid formulation, mannitol, auto-injector pen

## Abstract

Drug adherence is a significant medical issue, often responsible for sub-optimal outcomes during the treatment of chronic diseases such as rheumatoid or psoriatic arthritis. Monoclonal antibodies (which are exclusively given parenterally) have been proven to be an effective treatment in these cases. The use of auto-injectors is an effective strategy to improve drug adherence in parenteral treatments since these pen-like devices offer less discomfort and increased user-friendliness over conventional syringe-based delivery. This study aims to investigate the feasibility of including a monoclonal antibody as a solid formulation inside an auto-injector pen. Specifically, the objective was to evaluate the drug stability after a concentration (to reduce the amount of solvent and space needed) and freeze-drying procedure. A preliminary screening of excipients to improve stability was also performed. The nano-DSC results showed that mannitol improved the stability of the concentrated, freeze-dried antibody in comparison to its counterpart without it. However, a small instability of the C_H_2 domain was still found for mannitol samples, which will warrant further investigation. The present results serve as a stepping stone towards advancing future drug delivery systems that will ultimately improve the patient experience and associated drug adherence.

## 1. Introduction

Drug non-adherence is a major medical problem globally since the lack of positive outcomes in many treatments is often related to some degree of poor compliance or non-adherence. Drug adherence is, according to the World Health Organization “the degree to which the person’s behavior corresponds with the agreed recommendations from a health care provider”. Different types of intentional or non-intentional non-adherence can be distinguished, but in most cases, the outcome is an incorrect dosage schedule [1]. The probability of non-compliance increases for chronic treatment (only 50% of patients remain compliant), especially when the disease is silent or after symptom remission (e.g., rheumatoid arthritis and other autoimmune diseases). It has been estimated that up to 50% of treatment failures and up to 25% of hospitalizations can be ascribed to non-adherence in the U.S. [2,3]. The intervention of medical staff (direct, indirect, in-person, etc.) can improve drug adherence, but this is not always possible due to limited resources and in the case of home-based treatments. Therefore, drug adherence needs to be improved by other strategies such as patient education, regimen simplification, packaging optimization, minimization of adverse effects, and improved drug delivery devices [2].

It is widely known that oral or skin delivery are the most acceptable routes for drug administration. Nevertheless, these routes are not always possible, as is the case of proteins such as monoclonal antibodies, whose use in therapeutics has increased exponentially in recent years [4,5,6]. For these biomolecules, parenteral administration is essential to guarantee the maximum therapeutic effect. The major disadvantage of these therapies and thus, the main cause of compromised adherence, lies in the fact that a subcutaneous injection is required. Normally, this entails administration by a healthcare professional, but travel to the healthcare center and the common fear of needles (trypanophobia) are amongst the main contributors to non-compliance in this kind of treatment. Technology can be a reliable ally in this regard. There is clear evidence that the use of self-injections may increase treatment adherence when compared to injection by the healthcare workers [7]. Moreover, auto-injectors are self-injection devices that automatically insert the needle and deliver the required dose, thus providing even more benefits in terms of drug adherence: less discomfort and fewer adverse effects, increased user-friendliness (no need to handle/prepare needles, solvents, etc.), portability and flexible scheduling [7,8,9].

On the other hand, self-injector and auto-injector devices are not exempt from limitations: precise conditions for storage are sometimes required, appropriate reconstitution of the active substance must take place before injection, and limited customization or reuse is possible. Furthermore, these devices generally lack ‘intelligence’, and do not offer a patient/clinician interface, programmability, misuse prevention (e.g., incomplete delivery), or wireless connectivity. Furthermore, although solid (i.e., freeze-dried) formulations are known for their ability to keep the biological components stable for longer, current auto-injectors cannot easily deliver solid formulations. Instead, complex rehydration procedures before injection or high-cost fabrication processes are required, which compromise the usability and final price of the product.

In this first pilot study, we investigate the feasibility and potential advantages/limitations of including a biomolecule as a solid formulation inside an injector pen. The focus of this article lies on evaluating the stability and compatibility of the drug inside a new housing, though it is important to acknowledge that the origin of this study stems from a broader project (*Moore4Medical*)—the design and development of a high-tech, wirelessly-connected injector pen. The present investigation serves as an essential stepping stone towards advancing the field of pharmaceutical delivery systems, potentially providing healthcare professionals and patients with a user-friendly and efficient method of administration.

With these premises, this study will focus on exploring the feasibility of including a biomolecule in this new delivery system. A commercial monoclonal IgG_1_ antibody (ADA) will be used as a model drug since it is one of the most commonly used IgG subtypes in therapeutics, which makes the possible findings of this pilot study highly transferable. Since the final aim is to host it inside an injector pen in a solid state, a lyophilization step is required. Monoclonal IgG_1_ antibodies often require considerable amounts of liquid during their injection, therefore requiring the use of bigger and less manageable devices. These factors have also been reported to hinder treatment adherence [10]. Because of that, a concentration procedure to reduce the amount of solvent and space needed was studied. Its stability after lyophilization and concentration will be explored through Differential Scanning Calorimetry (DSC), in particular through “nano-DSC” or “nano-differential scanning calorimetry”. This type of DSC is designed to measure energy transitions in an ultra-sensitive manner, enabling the study of diluted in-solution biomolecules. For the particular case of proteins, nano-DSC is of special interest, since it allows the study of thermal transitions of liquid samples with good sensitivity and resolution. This technique offers a full thermodynamic profile of the biomolecules’ thermal events (protein unfolding, melting, denaturation, hydrolysis, among others). As it is widely known, the structure of biomolecules such as monoclonal antibodies is crucial for their correct functioning, therapeutic activity, and safety. Therefore, monitoring their stability is a key factor during drug development, for which the use of nano-DSC is a highly suitable technique.

## 2. Materials and Methods

### 2.1. Materials

Monoclonal antibody IgG1 type (adalimumab, ADA) was kindly provided by AbbVie (Cork, Ireland). The qualitative composition of ADA formulation includes mannitol, citric acid monohydrate, sodium citrate, disodium phosphate dihydrate, sodium dihydrogen phosphate dihydrate, sodium chloride, and Polysorbate 80. The samples were in their commercial liquid form (100 mg/mL) and preserved at 4 °C until use. The osmolarity of the original formulation was measured by a Cryoscopic Osmometer (OSMOMAT^®^ 030, Gonotec^®^, Germany). This equipment measures the freezing point depression, thus determining the total osmolality of aqueous solutions. The osmolarity of commercial ADA was 0.274 ± 0.0175 Osm/L and possessed a pH of 5.3 ± 0.007. D-mannitol, D-trehalose, and polyethylene glycol 6000 and 8000 (PEG) were purchased from Sigma-Aldrich (Burlington, USA) and Dextran 40 kDa was obtained from Pharmacosmos (Holbæk, Denmark). These ingredients were explored as potential excipients to improve the stability of the freeze-dried, concentrated IgG_1_ samples.

### 2.2. Monoclonal Antibody Quantification

ADA quantification was performed by spectrophotometry in a SimpliNano device (GE Healthcare, Belfast, UK). For that purpose, a calibration curve of ADA was constructed by diluting the commercial antibody in ultrapure water, obtaining 9 concentrations ranging from 0.1 to 5 mg/mL (R^2^ = 0.996), each concentration measured in triplicate.

### 2.3. Nano Differential Scanning Calorimetry

Thermal analyses were carried out by means of a TA Instruments Nano-DSC (WatersTM, New Castle, DE, USA; model 602000), equipped with a continuous capillary sample cell (0.3 mL). The analysis method was established between 40–100 °C with a heating rate of 1 °C/min. All the samples were diluted (ultra-purified water, 1 mg/mL concentration), quantified as previously described (Section 2.2), degassed for 10 min in a vacuum (at 490 mmHg) to eliminate microbubbles, and directly subjected to thermal analysis. Prior to any nano-DSC analysis, a water-water scan was conducted both to check the suitability of the instrument as well as to obtain the instrument baseline.

The thermograms were studied through the NanoAnalyze software (TA Instruments, Newcastle, USA), which provides an empirical approach and fits the thermograms with multiple Gaussians after the deconvolution of the individual domains. Due to the intrinsic properties of the antibody unfolding (multi-state process), the Voigt model was selected among all of the fittings offered by the software. Voigt is a hybrid of a Gaussian and Lorentzian function and is appropriate for peak deconvolution and transitions that are not a true two-state unfolding event, as happens for complex proteins such as IgG antibodies [11,12,13]. Before deconvolution, manual integration baselines (polynomial) were applied in all cases.

### 2.4. Dynamic Light Scattering and Zeta Potential

The hydrodynamic size and effective charge of antibody in ultra-purified water (milliQ water) solutions were measured with the use of a Malvern Zetasizer Nano Series (Malvern Instruments, Worcestershire, UK) equipped with a 4 mW, 633 nm laser. The hydrodynamic size of ADA was determined by Dynamic Light Scattering (DLS), working with disposable DTS0012 cuvettes and performing 100 scans/measurements. The ζ-potential was measured in disposable DTS1070 cells using 30 s of delay between measurements. All measurements were performed in triplicate after 120 s of equilibration.

### 2.5. Preliminary Studies: Factors Influencing Nano-DSC Results

A batch of preliminary studies was performed to understand how different stressing factors will affect the monoclonal antibody and how they affect the ADA thermogram. To study the dependence between analyte concentration and thermogram result, three dilutions of commercial ADA were prepared (0.5 mg/mL, 1 mg/mL, and 1.5 mg/mL) and analyzed through nano-DSC. Additionally, temperature and denaturants were employed as forced degradation agents. The influence of temperature was assessed by subjecting ADA (1 mg/mL) to room temperature (RT), 40 °C and 80 °C for 24 h in dark, static conditions. The strong salt Guanidine hydrochloride (GdnHCl) was also combined with diluted ADA at two different concentrations (0.005 and 0.05 M). All these samples were then analyzed through nano-DSC, DLS, and ζ potential. The final aim was to understand the degree of impact of these scenarios over the ADA thermogram under the working conditions of this study.

### 2.6. Solid Monoclonal Antibody Approaches

For the housing of a monoclonal antibody in solid form inside an auto-injecting system, it is necessary to study the stability of the drug after a freeze-drying process and its concentration to reduce the volume and space needed within the device.

The freeze-drying process was performed in a Telstar Lyobeta equipment (Terrassa, Spain). The lyophilization recipe consisted of freezing the samples to −50 °C (1 °C/min) and maintaining this temperature for 2 h. Then, 2-step primary drying was performed at a pressure of 0.2 mBar during the whole process. The first primary drying step occurred at −50 °C for 5 h (first step) and at 20 °C for 6 h (second step), by ramping up at 0.5 °C/min. Secondary drying at 20 °C lasted for another 24 h. The influence of the freeze-drying process (FD) was explored through nano-DSC.

ADA formulations were concentrated by means of Vivaspin^®^ 500 centrifugal concentrators, with a cut-off membrane of 5000 MW (note that ADA monoclonal antibody has a 148 kDa of MW). The device was subjected to 25,155 G for 45 min at a constant temperature of 25 °C. The resultant concentrated ADA samples are abbreviated as “C-ADA”. The osmolarity of C-ADA samples was determined (Gonotec^®^, OSMOMAT^®^ 030) and compared with ADA, confirming the loss of solutes in the commercial formulation due to the membrane cut-off. To determine osmolarity, C-ADA was diluted in ultra-purified water to 1 mg/mL. Highly concentrated antibodies tend to suffer from instability, especially considering that part of the excipients of the original liquid formulation will be lost during this process. To counteract this effect, different excipients were used. Each of these ingredients were added to C-ADA before or after the concentration process, depending on their molecular weight: D-mannitol (Man) and D-trehalose (Tre) were added to C-ADA after the concentration process, whereas dextran 40 kDa and polyethylene glycol (PEG 6000 and 8000) were added beforehand since they had >MW than the vivaspin devices (5 kDa MWCO). Different concentrations of each excipient were explored and their selection as possible stability enhancers was based on nano-DSC results.

### 2.7. Stability Pilot Study

Based on the results of the excipient screening, a pilot study was designed to explore the stability of the concentrated, solid ADA formulations with and without the selected excipient (Man). To do so, freeze-dried (FD), concentrated samples of ADA with mannitol (C-ADA FD Man) and without it (C-ADA FD) were preserved at 4 °C and room temperature (RT) for 6 months and compared with the stability of non-concentrated, freeze-dried ADA (ADA FD) preserved under the same conditions. Prior to any other analysis, freeze-dried samples were reconstituted and diluted (when needed) in ultra-purified water.

### 2.8. Viscosity Studies

The rheology profile of the selected formulations (C-ADA, C-ADA FD, C-ADA Man vs. ADA) was explored by performing rotational flow curves from 1–1000 1/s in an AR Rheometer (TA Instruments) equipped with a plate/plate sensor (φ 20 mm). The temperature was maintained constant at 25 °C during the whole experiment. The gap used was set at 600 nm (thus containing 200 µL/sample). All the analyses were performed in triplicate. 

## 3. Results and Discussion

### 3.1. Preliminary Studies: Factors Influencing Nano-DSC Results

**Figure 1:**
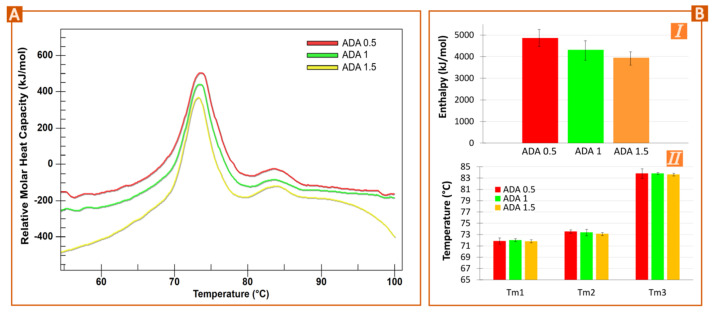
Effect of ADA concentration in nano-DSC results (**A**,**B**). (**A**) Original nano-DSC thermograms for 0.5 to 1.5 mg/mL of ADA diluted in water. “Relative molar heat capacity” was used in the *y*-axis since the thermograms have been manually shifted in height to allow easier comparisons. (**B**) Results obtained after deconvolution of nano-DSC thermograms using Voigt model (**I**) total enthalpy and (**II**) melting temperature of the three ADA unfolding events.

The thermograms of different ADA concentrations (0.5, 1, and 1.5 mg/mL) are gathered in Figure 1A. As expected, the three samples gave rise to parallel thermograms showing the same thermal events in the same position. Monoclonal IgG antibodies are known to possess three thermal transitions that can be overlapped in different degrees [11,14,15]. In this particular case, ADA possesses two visible thermal transitions at 74 °C and 84 °C. The main event corresponds to the constant heavy chain-2 and the antigen-binding region unfolding (C_H_2 and Fab domains, overlapped), while the second corresponds to the constant heavy chain (C_H_3 domain), which is in agreement with the previous literature sources [11,16,17,18]. This overlapping is due to the close unfolding event of the C_H_2 fragment (72–74 °C) with the F_ab_ domain (73–74 °C) [16]. The thermograms’ deconvolution showed that the main temperature event slightly shifted toward lower temperatures as the concentration of the antibody increase, something that was confirmed by model fitting (see Figure 1B, II). The total enthalpy values also decreased as the ADA concentration rose (from 0.5 mg/mL to 1.5 mg/mL). Higher concentrations can lead to higher interactions between ADA molecules and so, to higher aggregation phenomena and/or higher cooperative unfolding, thus needing less energy per mole to unfold. When it comes to IgG antibodies, C_H_3 or C_H_2 are the domains reporting aggregation-prone behavior [13,19,20]. Normally, “the least stable domain is involved in the aggregation process” [13], which means that for ADA it would be the C_H_2 domain. In any case, it has been demonstrated that enthalpy reductions are related to losses of native antibody structure, which sometimes has been associated with aggregation events [21,22]. The hydrodynamic size and ζ potential of the ADA dilutions were also determined. The results of the DLS analysis were inconclusive, (data not shown for simplicity), but the zeta measurements did report a progressive decrease in positive net charge (16.9 ± 0.5, 13.65 ± 1.224, 8.43 ± 0.63 mV from 0.5 mg/L to 1.5 mg/mL), which implies lower intermolecular repulsions and so, easier interactions and faster unfolding process.

**Figure 2:**
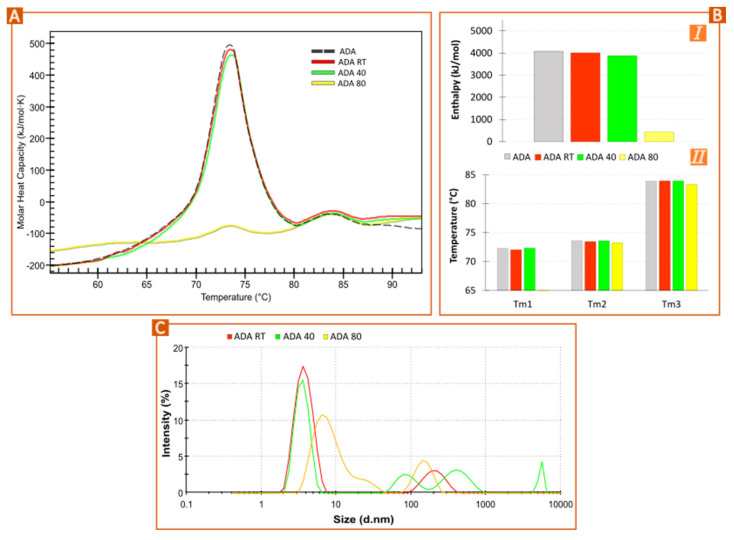
Forced degradation: ADA temperature effect. (**A**) Nano-DSC thermograms of diluted ADA (1 mg/mL) subjected to different temperatures (RT or “room temperature”, 40 and 80 °C) for 24 h. (**B**) Results obtained after thermograms deconvolution using the Voigt model: (**I**) total enthalpy; (**II**) melting temperatures of the three ADA unfolding events. (**C**) Profile of hydrodynamic particle size of ADA RT, ADA 40, ADA 80.

Three ADA solutions were maintained at RT, 40°, and 80 °C for 24 h (samples codified as ADA RT, ADA 40, and ADA 80). The results, gathered in Figure 2, showed that this stressful factor triggered a degradation process that is detected through nano-DSC by enthalpy reduction. Since ADA onset temperature (T_onset_) is around 65 °C, it is expected that neither ADA RT nor ADA 40 were significantly affected by these temperatures, at least for short times. The T_onset_, in this case, is the temperature at which the sample starts unfolding at a measurable rate. Compared to commercial ADA (Figure 2A, black, dashed line), the samples at RT and 40 °C suffered a slight decrease in height (Figure 2A, red and green lines, respectively), confirmed by the calculated total enthalpy after deconvolution (Figure 2B,I). This result indicates that nano-DSC can be a suitable technique to monitor ADA structural stability for temperatures below the T_onset_. Nevertheless, the lower the temperature, the lower the difference between thermograms, which could be misleading if no other results are available. On the other hand, for ADA 80 (Figure 2A, yellow line), the first thermal event practically disappears, as the temperature of stress is >T_onset_. It is also worth mentioning that the enthalpy decrease affects the first thermal event (C_H_2 and F_ab_ domains), with the C_H_3 not showing changes in height (Figure 2A) for any sample.

The transition midpoint temperatures (T_m1_, T_m2_ and T_m3_) were constant for ADA, ADA RT, and ADA 40 (Figure 2B,II). By looking at the thermogram of ADA 80 (Figure 2A, yellow line), the C_H_3 thermal event was practically superimposed over the others. Due to the small size of the thermal event in the ADA 80 thermogram [23]

From these results, it is possible to conclude that temperature-stress conditions for ADA translate into an enthalpy decrease with no significant changes in transition midpoints when analyzed by nano-DSC. It has already been reported that temperature-stress conditions induce ADA aggregates of different MW and solubility [22,24]. Wu et al. stated that aggregation is temperature dependent, having an Arrhenius regime for T ≥ T_m1_ (C_H_2 transition midpoint) or a non-Arrhenius regime for T < T_m1_ [25]. This particle aggregation was confirmed by DLS measurements (Figure 2C). The particle profiles and mean size of ADA RT and ADA 40 are similar to the dimension profile of ADA already reported in the literature [26], although the differences can be attributed to the sample composition and experimental conditions. ADA 80 reported a clear increase in particle size, as shown by the shifting of the signal toward higher diameters.

**Figure 3:**
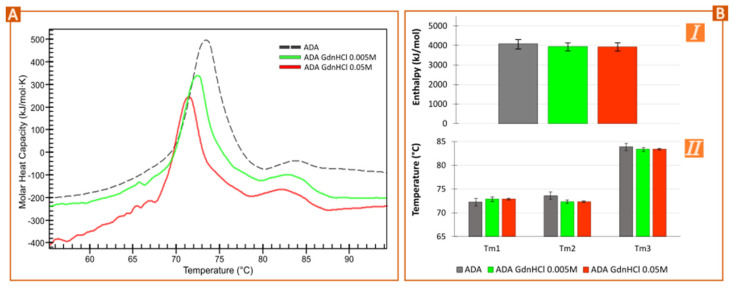
Forced degradation: effects of GdnHCl denaturant. (**A**) Nano-DSC thermograms of ADA (1 mg/mL) diluted in GdnHCl 0.005 M and 0.05 M compared with the commercial formulation. (**B**) Total enthalpy (**I**) and temperature midpoints (**II**) obtained after deconvolution of the thermogram’s samples (Voigt model).

The addition of GdnHCl to ADA reduced both the midpoint temperature (leftward shifting of the thermogram) and enthalpy values (Figure 3A,B) [12,13]. This effect was directly proportional to GdnHCl concentration. Some researchers have reported that the GdnHCl denaturing effect is related to the increase of water surface tension and the water replacement from the proteins’ first solvent shell after GdnHCl binding to the protein backbone and side chains [27]. More specifically, strong electrostatic interactions between guanidine and charged peptide side chains were reported by Monera et al. [28]. The direct interactions between GdnHCl and proteins have been confirmed, especially with aromatic side chains of amino acids [29]. A more recent study by Garidel et al. reports that the direct binding of guanidine with proteins is an exothermic event that compensates the endothermic unfolding, which would explain the enthalpy reduction of ADA (Figure 3B,I). They also stated that, according to the stoichiometry GdnHCl-to-aminoacids for the small and the large domain, the C_H_2 domain would be more accessible to the denaturant and easier to destabilize [12]. In this regard, it is worth pointing out that the mathematical fitting and the thermogram’s deconvolution (gathered in Figure S1) are contradictory to these statements. Based both on the existing literature and on the deconvolution of commercial ADA samples, it was determined that the Ada’s C_H_2 and F_ab_ domains unfolding events overlapped, the C_H_2 one happening at lower temperatures than F_ab_ [11,16,17]. The addition of GdnHCl reports that the C_H_2 domain remains unaffected, the F_ab_ one suffering a shift toward lower temperatures (Figure S1). This result is inconsistent with the current literature, which states that it is easier to destabilize C_H_2 by guanidine [12,13]. These differences could be ascribed to the differential GdnHCl concentration used: the interactions between GdnHCl and ADA at low concentrations are different and they are mainly ascribed to the F_ab_ domain rather than to C_H_2 [13].

As well as structural modifications, the presence of GdnHCl led to changes in the hydrodynamic size of ADA and ζ-potential values (Figure S2). Strong electrostatic interactions between GdnHCl and charged peptide chains have been reported [13,28], which could explain the ζ-potential in Figure S2. Moreover, the presence of low concentrations of GdnHCl can trigger partial unfolding and so, aggregation [13,30].

### 3.2. Solid Monoclonal Antibody Approaches

**Figure 4:**
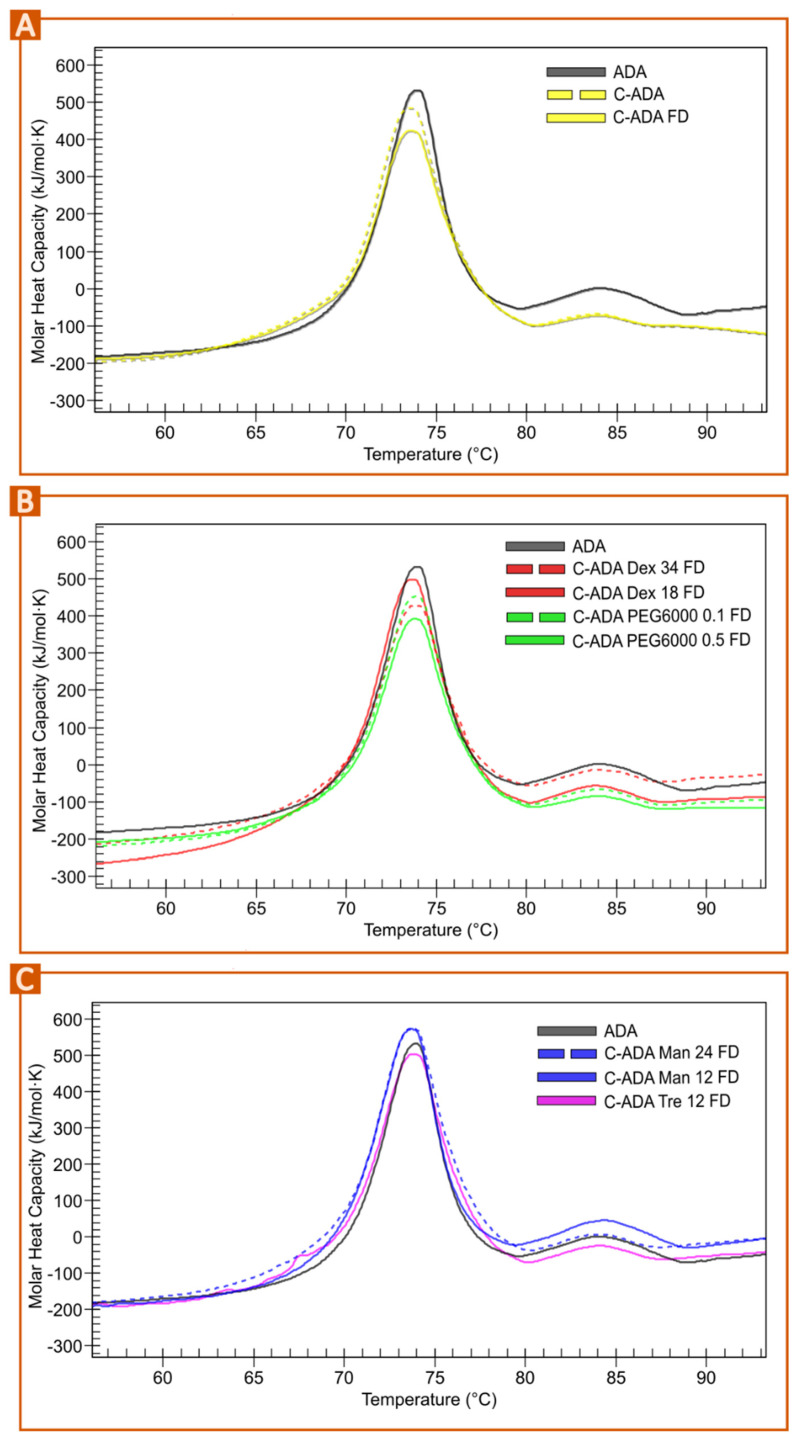
Nano-DSC results of concentrated ADA before freeze-drying (C-ADA) and after it (C-ADA FD) in comparison with the thermogram of ADA (**A**). Thermograms of freeze-dried C-ADA after the addition of different excipients: Dex and PEG 6000 (**B**) and Man, Tre (**C**).

The concentration and lyophilization process of ADA is expected to trigger instability of the molecule due to the (i) increased antibody concentration, hypothetically leading to easier unfolding due to closer protein-protein interactions, as proved in Figure 1, and (ii) removal of excipients during concentration. After the concentration process, the original ADA concentration (100 mg/mL) increased up to 234.55 ± 54.85 mg/mL. The osmolarity values reduced from 0.274 Osm/L for ADA (100 mg/mL) to 0.150 Osm/L for C-ADA (100 mg/mL), proving the loss of part of the excipients during the concentration procedure. The thermograms of both C-ADA before and after lyophilization (C-ADA FD, Figure 4A) show a decrease in enthalpy in comparison to ADA. Additionally, a small shift toward lower temperatures can be observed for these samples versus the commercial formulation, especially around 65–70 °C. This shoulder could be related to a shift of the C_H_2 fragment toward smaller temperatures and so an instability of this particular fragment. The need to include other excipients in the concentrated formulation is therefore clear, the primary objective being the improvement of the monoclonal antibody stability after the concentration and freeze-drying step.

Different PEGs have been successfully used as cryoprotectants of solid formulations, proteins, and even cells [31,32,33,34]. In most cases, high molecular weight PEGs (from 2000 to 10,000) are preferred for cryoprotection. In view of these promising results, PEG 6000 and 8000 were selected as possible candidates as C-ADA cryoprotectants to be added before the concentration process. Different PEG concentrations were tested (from 0.1 to 5% *w*/*w*), though only 0.1 and 0.5% could be successfully added to C-ADA. The addition of PEG 6000 and 8000 did not show any improvement in C-ADA thermal stability after freeze-drying. More precisely, PEG 8000 caused instability of the antibody during the concentration process, since the sample acquired a turbid/translucent appearance (data not shown), so they were immediately discarded from the study. PEG 6000 samples (at 0.1 and 0.5% *w*/*w*) were concentrated without macroscopic instability evidence, though the nano-DSC revealed a significant enthalpy loss (Table 1, Figure 4B), whereupon these samples were discarded.

Samples with Dex 34 mg also showed enthalpy losses (Table 1), while Dex 18 mg revealed a more stable sample but with the shoulder between 65–70 °C (Figure 4B). Moreover, reconstitution of C-ADA Dex 34 FD and C-ADA Dex 18 FD took the longest time, a reason why these excipients were also discarded at this point of the study. Dex is an effective cryoprotectant and has already been used in mAb formulations, though the stability improvement of the protein was limited [35], which is in agreement with our results. The higher stability was found for the lower Dex concentration (16.8 mg). In this study, the addition of 18 mg of Dex reported no enthalpy losses (Table 1), at first sight indicating proper stability. Nevertheless, the detailed study of the thermogram and the subsequent fitting and deconvolution revealed that the unfolding event for the C_H_2 domain decreased in temperature, from 72.7 °C in ADA to 68.4 °C in C-ADA Dex 18.

**Figure 5:**
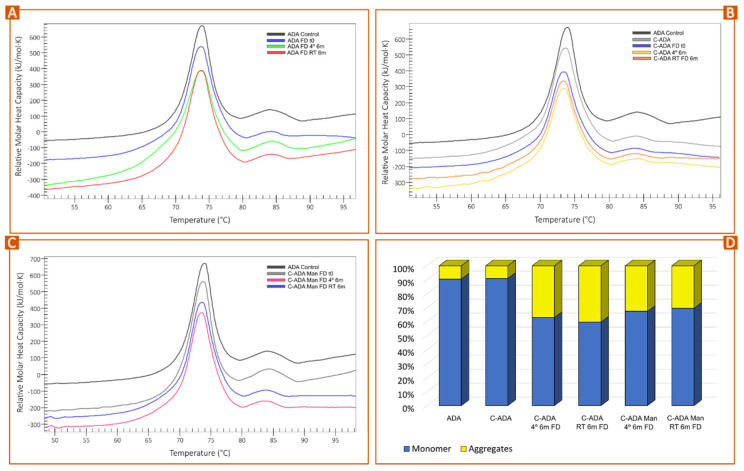
Time evolution of ADA (**A**), C-ADA (**B**), and C-ADA Man (**C**) thermograms before and after freeze-drying (FD) and after 6 months at room temperature (RT 6 m) and at 4 °C (4° 6 m). A random replicate per sample was used for this figure. (**D**) Monomer and aggregate content obtained by means of DLS (mean values; n = 3). Results of ADA FD samples after 6 months are not included for simplicity.

Sugars are the most widely known excipients for cryoprotection and protein stability, among which Tre and Man can be highlighted [31,35,36,37,38,39,40,41]. These excipients, due to their low MW, were added to C-ADA after the concentration process. The addition of Tre and Man was tested at two different concentrations (24 and 12 mg/mL). The concentration of mannitol was selected according to the composition of commercial adalimumab (containing 12 mg/mL of Man, see Section 2.1). Double this concentration (24 mg/mL) was also tested, based on the existence of other IgG_1_ FDA-approved formulations using even higher ones [42] These two ingredients soften the enthalpy losses of C-ADA in comparison with ADA (Figure 5C, Table 1). Studying the results in more detail, it is possible to observe that Tre 12 and Man 24 produced a slight shift of the thermogram toward lower temperatures (see 65–70 °C range) (Figure 4C) and a reduction of AUC of the C_H_3 domain (80–90 °C) which were not present in C-ADA Man 12. (C-ADA Man 12 FD is the one that least alters the ADA thermogram, with less enthalpy loss and no shoulder found between 65–70 °C. It seems that C-ADA Man 12 FD shows an increased stability in comparison to ADA as shown by the height of the unfolding events (Table 1, Figure 4C). As a consequence of the above, C-ADA Man 24 and C-ADA Tre were not included in the pilot study. According to Haeuser and co-workers “mannitol does not contribute to protein stability due to its crystalline nature requiring a certain amount of amorphous stabilizer” [35]. It is known that Man is prone to crystallize as mannitol hemihydrate (MHH) if the freeze-drying conditions are not optimal (i.e., if the secondary drying does not eliminate 100% of residual water). The water released by MHH during storage has the potential to cause instability [39,43]. This can be solved by subjecting the freeze-dried sample to a more aggressive secondary drying (≥40 °C), which would be detrimental for the major part of the biomolecules, such as in the case of ADA. In other words, this is not possible in this particular case and the secondary drying was performed at 20 °C in order to guarantee the stability of the antibody. In a more detailed study, Man was used as the cryoprotectant of a monoclonal antibody in combination with sucrose [38]. This study revealed that “the limiting factor for the implementation of Man as a crystalline bulking agent in the lyophilized mAb-based formulation is not the presence of MHH but rather the content of the amorphous stabilizer and its molar ratio to protein” [38]. The authors also observed that increasing concentrations of monoclonal antibodies can inhibit the formation of MHH during lyophilization. The present results indicate that Man 12 was able to increase C-ADA stability, at least for short times after lyophilization. According to this statement, the high concentration of C-ADA could prevent the formation of MHH and, subsequently, improve the long-term stability of the C-ADA Man FD system concerning the ADA counterpart.

In view of the aforementioned results, C-ADA Man 12 were selected as the sample with higher stability and, therefore, the most promising one for the development of new auto-injector hosting concentrated, solid monoclonal antibody formulations. The following steps of this study will be focused on exploring the stability of the solid for 6 months under different conditions.

### 3.3. Stability Monitoring of Solid Formulations

**Figure 6:**
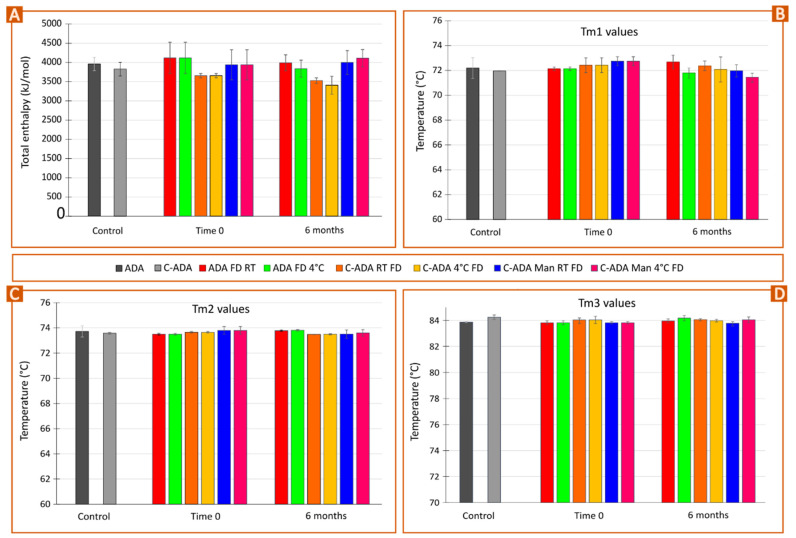
Pilot time stability study of non-concentrated (NC) and concentrated monoclonal antibody formulations (C-ADA) after freeze-dying (FD) and preservation at room temperature (RT) and at 4 °C in static conditions. C-ADA samples containing Man (C-ADA Man) were also freeze-dried and preserved at the same conditions. Bars gather the results obtained after fitting and deconvolution of thermograms (Voigt model): (**A**) total enthalpy values; (**B**) T_m1_ values; (**C**) T_m2_ values; (**D**) T_m3_ values. Mean values ± s.d. (n = 3).

Freeze-dried samples of commercial antibody (ADA FD) monitored for 6 months at 4 °C and RT (Figure 5A) showed no significant enthalpy changes. This result was supported by the fitting and deconvolution of each thermogram (Figure 6A). The sample at 4 °C (ADA FD 4° 6 m, green) showed the appearance of a shoulder between 65–70 °C, though the average result after fitting and deconvolution suggested this change is not significant (Figure 6B). In any case, this is interesting since this did not happen for the freeze-dried sample maintained at room temperature (ADA FD RT 6 m). Together with the fact that the total enthalpy of the sample at 4 °C (Figure 6A) was slightly lower than that of ADA FD RT 6 m, it shows that the stability of ADA FD 4° 6 m is more compromised and the C_H_2 domain is the fragment being affected. The melting temperatures of F_ab_-C_H_1 and C_H_3 domains (T_m2_ and T_m3_) remained constant during the whole study (Figure 6C,D).

In parallel, concentrated, freeze-dried samples preserved at 4 °C (C-ADA FD 4°) and at room temperature (C-ADA FD RT) reported a progressive decrease in total enthalpy (Figure 5B and Figure 6A) or, in other words, antibody instability. Once again, the sample C-ADA FD 4° reported a slightly higher decrease in total enthalpy with respect to C-ADA FD RT, but the variability of the results makes it difficult to discern their significance (Figure 6A). In previous sections of this manuscript, it has been demonstrated that the increase in antibody concentration does make the unfolding easier due to closer contact between molecules (see Figure 1B) as well as the potential reduction of antibody monomer content (Figure 5D). Therefore, higher thermal instability of C-ADA FD samples is to be expected. The main difference between C-ADA and ADA samples after 6 months is the antibody fragment affected: for C-ADA samples (Figure 5B), no shoulder was found around 65–70 °C and the deconvolution revealed no changes in T_m1_. A closer look at the thermograms (Figure 5B) revealed a slight shift of the main melting point (F_ab_-C_H_1 domain) to the left, which was also confirmed by the T_m2_ values after fitting and deconvolution (Figure 6C). In any case, it is difficult to discern the significance of this change.

Concentrated samples with Man reported no enthalpy losses after 6 months (C-ADA Man RT FD and C-ADA Man 4° FD), notwithstanding the preservation conditions (Figure 6A). Regarding the unfolding temperatures, C-ADA Man 4° FD 6 m reported a decrease in T_m1_ values, which did not happen for C-ADA Man RT FD 6 m (Figure 6B). This is a similar behavior to the one reported for ADA FD 4° 6 m, which showed the same total enthalpy but the T_m1_ values for C_H_2 domain unfolding decreased after 6 months. The common factor for both samples is the presence of Man: for ADA, mannitol was present in the original, liquid formulation whereas, for C-ADA Man, this excipient was added after the concentration process to increase the stability of the new solid formulation (C-ADA Man).

As mentioned in previous sections, some of the literature resources have reported that mannitol is prone to crystallize after FD, especially as a result of heat or moisture [43]. The residual moisture in the freeze-dried sample could induce MHH crystallization and ultimately cause protein instability [39,43]. To prevent that, a secondary FD step at high temperatures is advisable [43]. Nonetheless, the secondary drying during FD was set at low temperature (20 °C) to prevent protein from degradation, which means that MHH formation could be expected [44]. Apart from the freeze-drying conditions, other factors such as protein concentration can influence mannitol crystallization. In this regard, the literature shows inconclusive results, since various studies reported that high protein levels could either inhibit or promote MHH formation [38,44,45,46]. The quantification of MHH was outside of the scope of the present study, and therefore it is impossible to discern if a higher concentration of ADA prevents or induces MHH formation. Notwithstanding this fact, nano-DSC results demonstrated that C-ADA Man FD samples were more stable than C-ADA FD ones, disregarding the preservation conditions. It is also possible to hypothesize that the preservation of C-ADA Man samples and ADA samples at low temperatures induces some kind of instability, potentially ascribable to the formation of MHH since mannitol was the common ingredient between them. Although the deconvolution revealed no significant changes in enthalpy for C-ADA Man 4° FD and C-ADA Man RT FD vs. the control, a shift in T_m1_ values was reported, indicating some instability of the C_H_2 domain (Figure 6B). Even if the C_H_2 domain does not directly participate in the adalimumab mechanism of action, alterations in this domain can modify the Complement-Dependent Cytotoxicity activity [47,48]. With these premises, it is possible to conclude that mannitol is an effective excipient to protect C-ADA formulations, though further studies are still needed to prevent any kind of instability, including that of the C_H_2 domain.

### 3.4. Viscosity Studies

**Figure 7:**
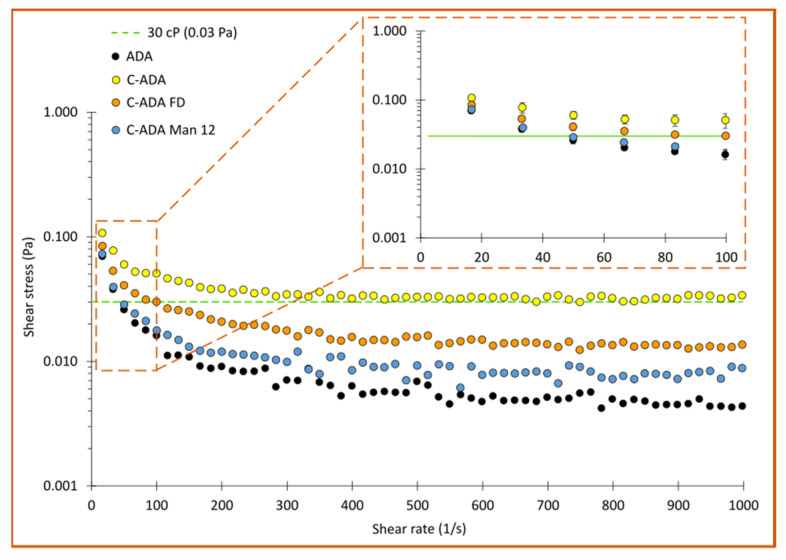
Apparent viscosities obtained from rotational flow curves (upward section) in a plate/plate geometry. The green, dashed line marks the viscosity value (0.03 Pa) established by the literature as critical for syringeability [49]. The enlarged curve offers a clearer vision of the low shear rate section for the sake of comparisons.

At high antibody concentrations, the viscosity of the final formulation can become the limiting factor for subcutaneous administration (due to difficult injection), and for the design and the proper functioning of the auto-injector. Several studies have determined that high protein concentration formulations can result in viscosities over 30 cP (0.03 Pa·s) [49,50,51]. The flow curves are gathered in Figure S3, while the apparent viscosity values obtained from flow curves are gathered in Figure 7. The viscosity of ADA (a commercial formulation containing 100 mg/mL of adalimumab) is in agreement with the one reported by Pathak and co-workers for a similar antibody concentration [52].

As expected, C-ADA (yellow dots, Figure 7) showed a remarkably higher viscosity in the whole range with respect to ADA (black dots). Taking as a reference the critical viscosity value reported by Roche et al. [49] (0.03 Pa), syringeability of the C-ADA formulation can be considered non-optimal (likely to cause some device complications and/or user inconveniences during injection), independently of the stress applied (η values > 0.03 Pa). Interestingly, this viscosity decreased after the freeze-drying and reconstitution (C-ADA FD, orange dots), and was even lower for the C-ADA Man sample (also after freeze-drying and reconstitution). The viscosity reduction in C-ADA FD vs. C-ADA could be related to a partial unfolding/denaturation of the protein during the freeze-drying process, as reported by the nano-DSC total enthalpy values of this sample vs. C-ADA and ADA (Figure 6A). In the case of C-ADA Man, the viscosity decrease is more interesting since the nano-DSC results did not show significant enthalpy losses or thermal instability signs. The C-ADA Man sample possessed very similar viscosity values at low shear rates (see the enlarged section of the curve), the difference becoming more noticeable at higher shear rates.

Sugars such as sucrose and mannitol have been reported to decrease the dielectric constant of solvents, thus increasing charge-charge repulsions between adjacent antibody molecules [53,54]. The preferential exclusion of sugars such as mannitol from the native structure of proteins has been reported to reduce reversible self-association of antibody molecules [41], something that could also explain the decrease in viscosity found for C-ADA Man.

In any case, since the viscosity values of C-ADA Man were practically identical to those of ADA from 10–100 1/s, and even if they increase from 100 1/s onwards, they are still significantly below the 0.03 Pa, viscosity values for which the subcutaneous administration starts to be problematic. This demonstrates that mannitol is also beneficial as an excipient in the formulation of highly concentrated ADA.

## 4. Conclusions

The development of a concentrated monoclonal antibody for inclusion as a solid formulation inside an injector pen, ultimately aiming to improve drug adherence, was attempted. Specifically, this manuscript focused on evaluating the influence of the concentration and freeze-drying processes on the stability of the drug. To reduce the amount of solvent and volume required inside the injector pen, the antibody formulation was also concentrated. Bearing in mind that the commercial formulation used here is already highly concentrated, a further concentration step and lyophilization are expected to hinder molecule stability, especially because part of the excipients in the liquid formulation will be eliminated during this process.

According to the pilot study and nano-DSC results, the addition of 12 mg/mL of mannitol to concentrated ADA before the freeze-drying process improved monoclonal antibody stability both at short and longer times (up to 6 months) in comparison to the concentrated counterpart without it. The stability profile of the concentrated sample with mannitol (C-ADA Man FD) was similar to the stability of the original commercial formulation (non-concentrated) after freeze-drying (ADA FD). In particular, the formulations with mannitol preserved at low temperatures (4 °C) reported instability of the C_H_2 fragment after 6 months. Since the common ingredient between C-ADA Man FD at 4 °C and ADA FD at 4 °C is the presence of mannitol, we hypothesize that this instability could be related to the formation of mannitol hemihydrate (MHH). Therefore, this formulation is not sufficient to guarantee the total stability of the said protein, and it is necessary either to either add other excipients to increase the stability of the C_H_2 fragment, or to optimize the freeze-drying process to prevent the formation of MHH. The addition of mannitol also reduced the viscosity of the formulation, which is of great interest to the scientific community that is recently focusing its efforts on formulating highly concentrated antibodies.

In conclusion, the introduction of monoclonal IgG_1_ antibody inside an auto-injector as a solid formulation is feasible in terms of molecular stability, after a proper optimization of the volume and formulation itself. The addition of mannitol demonstrated an improvement in the stability of a highly concentrated monoclonal antibody. Moreover, the viscosity values of this formulation suggest an adequate syringeability of the highly concentrated formulation. Although further studies will be required, these preliminary results indicate that it should be feasible to incorporate a formulation of this type inside an injector pen.

## Figures and Tables

**Table 1:** Comparison of total enthalpy values and maximum temperatures of ADA and C-ADA samples with and without excipients. The values of this table are extracted from the thermograms in Figure 4.

Sample	Total ΔH (kJ/mol)	T_m1_ (°C)	T_m2_ (°C)	T_m3_ (°C)
ADA	3959.78	72.78	74.02	83.83
C-ADA	3934.38	71.95	73.52	84.12
C-ADA FD	3515.73	71.97	73.61	84.37
C-ADA Man 24 FD	4091.45	68.80	73.67	83.77
C-ADA Man 12 FD	4130.36	72.60	73.68	83.72
C-ADA Tre 12	3885.89	68.50	73.80	83.59
C-ADA Dex 34 FD	3424.06	72.64	73.78	83.84
C-ADA Dex 18 FD	4097.09	68.45	73.54	83.80
C-ADA PEG 6000 0.1 FD	3247.79	72.98	73.78	83.98

## Supplementary Materials

The following supporting information can be downloaded at: https://www.mdpi.com/article/10.3390/pharmaceutics15082049/s1, Figure S1. (A) Mathematical deconvolution of ADA GdnHCl 0.005 M, (B) ADA GdnHCl 0.05 M and (C) commercial formulation, using Voigt model in all cases; Figure S2. (A) Hydrodynamic particle size (d.nm) and (B) ζ-potential (mV) of commercial sample (ADA), ADA GdnHCl 0.005 and 0.05 M; Figure S3. Flow curves (shear stress (1/s) vs shear rate (Pa)) of ADA, C-ADA, C-ADA FD and C-ADA Man 12 samples. All the samples possessed a thixotropic profile, the upper curve (10–1000 1/s having higher shear rate values than the donward-curve).
