# Supplementary material for: Stability of Monoclonal Antibodies as Solid Formulation for Auto-Injectors: A Pilot Study
Source: Pharmaceutics. 2023 Jul 30;15(8):2049. doi: 10.3390/pharmaceutics15082049 (PMC10459033; doi:10.3390/pharmaceutics15082049)
Supplement: Supplementary file 1 [file pharmaceutics-15-02049-s001.zip › pharmaceutics-2502656-supplementary.pdf]

Supplementary Materials

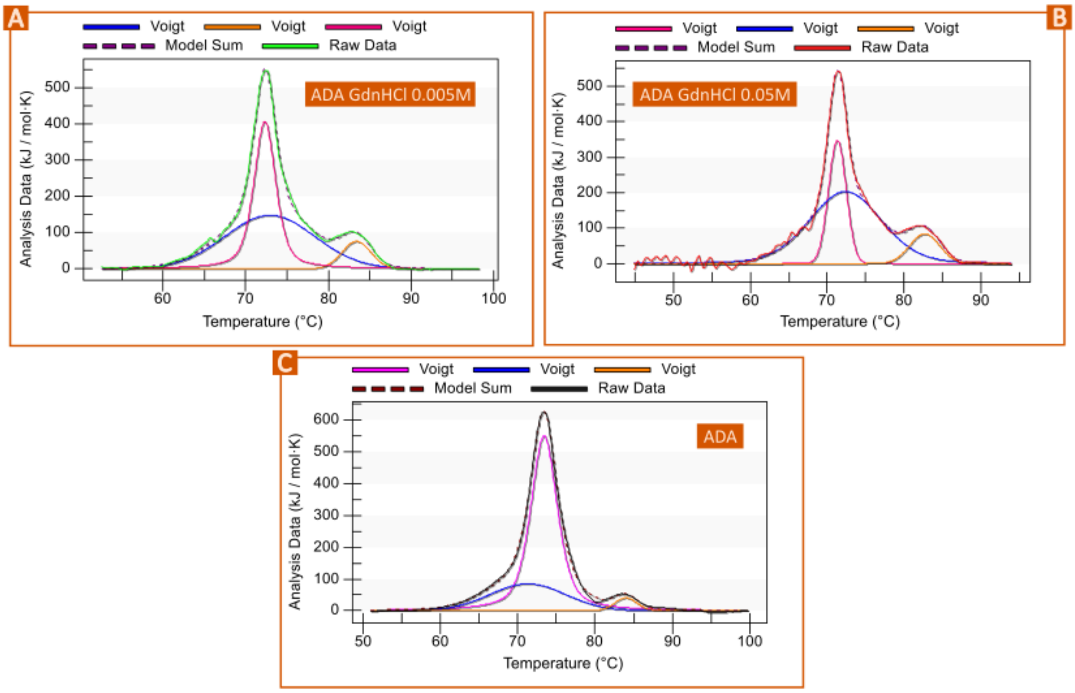

Figure S1. A) Mathematical deconvolution of ADA GdnHCl 0.005M, B) ADA GdnHCl 0.05M and C) commercial formulation, using Voigt model in all cases.

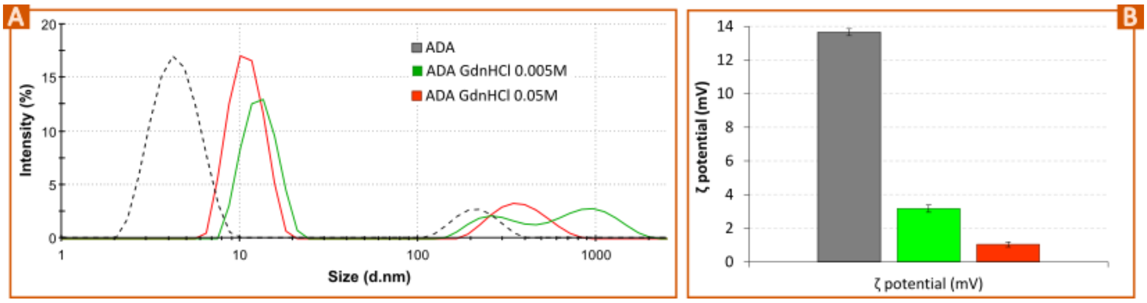

Figure S2. A) Hydrodynamic particle size (d.nm) and B) ζ-potential (mV) of commercial sample (ADA), ADA GdnHCl 0.005 and 0.05 M.

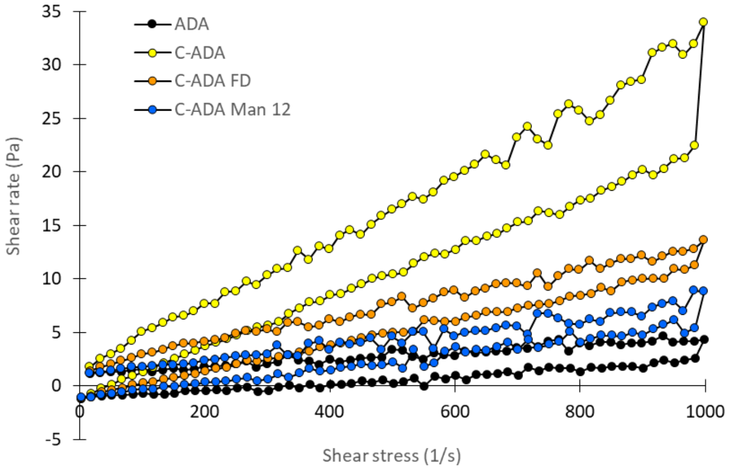

Figure S3. Flow curves (shear stress (1/s) vs shear rate (Pa)) of ADA, C-ADA, C-ADA FD and C-ADA Man 12 samples. All the samples possessed a thixotropic profile, the upper curve (10-1000 1/s having higher shear rate values than the donward-curve).
